# Baicalein blocked gastric cancer cell proliferation and invasion through modulated platelet type 12-lipoxygenase

**DOI:** 10.22038/ijbms.2024.80479.17422

**Published:** 2024

**Authors:** Jing Ye, Dan Qiao, Yingying Zhang, Yingshi Piao, Jingchun Jin

**Affiliations:** 1 Cancer Research Center, Yanbian University Medical College, Key Laboratory of Pathobiology (Yanbian University), State Ethnic Affairs Commission, Research and Innovation Group of Yanbian University, Yanji 133002, China; 2 Department of Anatomy, School of Basic Medicine, Binzhou Medical University, Yantai, 264000, China; 3 Department of Pathology, Sir Run Run Shaw Hospital, Zhejiang University, Hangzhou, 310000, Zhejiang, China; 4 Chifeng Municipal Hospital, Chifeng, 024000, Inner Mongolia, China; 5 Department of Internal Medicine, Yanbian University Hospital, Yanji 133000, China; # These authors contributed equally to this work

**Keywords:** Apoptosis, Baicalein, Epithelial mesenchymal – transformation, ERK1/2, Gastric cancer, Platelet type 12-lipoxygease

## Abstract

**Objective(s):**

Baicalein (BAI) is one of the main ingredients of *Scutellaria baicalensis *georgi. Its pharmacological effects have been widely reported in various cancers. However, the specific molecular mechanism of BAI in gastric cancer (GC) has not been defined. This study investigates BAI’s inhibitory effect on gastric cancer and its potential mechanisms.

**Materials and Methods:**

Gastric normal (GES-1 cells) and cancer cells (MKN-74 and MGC-803 cells) were treated with different concentrations of BAI. Cell proliferation and migration were assessed by MTT, colony formation, wound healing, and transwell assays. Flow cytometry and Hoechst 33342 staining were used to detect the cell apoptosis. IF and WB tests were employed to detect EMT-related protein. Finally, the anti-tumor effects of BAI were verified in *in vivo* xenograft models.

**Results:**

Our results show that the cell viability of MKN-74 and MGC-803 cells was significantly decreased in a time- and dose-dependent manner after BAI treatment by MTT assay. The expression levels of *p*12-LOX genes, which were determined by quantitative RT-PCR and WB, in MKN-74 cells were higher than those in GES-1 cells. As shown by the wound healing assay and Transwell assay, the treatment with BAI also significantly suppressed GC cell migration and invasion. Besides, BAI inhibited the phosphorylation of ERK1/2 and MEK1/2 in GC cells, as revealed by WB. Furthermore, BAI significantly inhibited tumor growth capacities in a xenograft model.

**Conclusion:**

BAI shows a significant anti-tumor effect and inhibition on tumor cell migration and invasion, which is probably through regulation of *p*12-LOX modulated epithelial-mesenchymal transformation.

## Introduction

Gastric cancer (GC) is a common malignant tumor of the digestive system with high mortality and morbidity worldwide (1). The metastasis may lead to treatment failure (2). Epithelial-mesenchymal transformation (EMT) is closely related to the tumor metastasis (3). During EMT, the actin cytoskeleton is reorganized, and cell-matrix contacts are increased, leading to the dissociation of tumor cells from the surrounding cells and the enhancement of tumor migration and invasion (4). Targeting the proteins involved in the EMT process is an important measure for cancer treatment, including gastric cancer. The role and mechanism of Chinese herbal medicine in cancer metastasis have attracted much attention (5). Baicalein (BAI) is a phenolic flavonoid compound extracted from ingredients of *Scutellaria baicalensis georgi*. It has anti-bacterial, anti-inflammatory, anti-oxidant, antiviral, and anti-allergic activities (6) and anti-tumor effects on thyroid, melanoma, breast, ovarian, and other cancer cells (7-10). Additionally, BAI is a selective inhibitor of Platelet type 12-lipoxygenase (*p*12-LOX) and has low toxicity and side effects (11). Ma *et al.* found that BAI suppresses metastasis of breast cancer cells by inhibiting EMT (12). However, the role and mechanism of BAI in gastric cancer have not been fully revealed. *p*12-LOX is a key enzyme in the conversion of arachidonic acid to 12-hydroperoxyeicosatetraenoic acid (12-HETE). *p*12-LOX was first found in platelets and in mammalian epidermal and tumor cells (13). It has been reported that 5-LOX and *p*12-LOX can promote tumor cell proliferation and the incidence of breast cancer, esophageal squamous cancer, hepatocellular carcinoma, pancreatic cancer, *etc.* (14-17)*.* On the contrary, 15-LOX, 8-LOX, epidermis-type 12-LOX (*e*12-LOX), and leukocyte-type 12-LOX (*l*12-LOX) play inhibitory roles in tumor development (18). Chen *et al.* found that 12-LOX promoted the proliferation of gastric cancer AGS cells and inhibited their apoptosis, indicating that there is a close relationship between 12-LOX and gastric cancer development (19).

In this study, the effects of BAI on gastric cancer cells were investigated. The expressions of *p*12-LOX, EMT-related protein markers (E-cadherin, Vimentin, Snail, *etc.*), and proteins in ERK1/2 signaling pathways were analyzed. The underlying mechanism of BAI in gastric cancer cells was analyzed and discussed. 

## Materials and Methods

***Cell lines and cell culture***

The GES-1 normal gastric mucosa cell line, MKN-74, and MGC-803 human gastric cancer cell lines were supplied by Yanbian University Cancer Research Center (Yanbian, China). All cell lines were cultured in RPMI-1640 medium (Gibco BRL, Grand Island, NY, USA) supplemented with 10% fetal bovine serum (FBS) and 1% penicillin/ streptomycin (Gibco BRL, USA) at 37 °C in a humidified atmosphere with 5% CO_2_. MKN-74 cell line was characterized by Genetic Testing Biotechnology Corporation (Suzhou, China) using short tandem repeat (STR) markers. 

***Antibodies***

Antibodies against cleaved caspase-3 (#9661), -8 (#9496), -9 (#20750), p-MEK-1/2 (#2338), and p-ERK1/2 (#9101) were purchased from Cell Signaling Technology (Boston, USA). Antibodies against *p*12-LOX (ab211506), E-cadherin (ab152102), ZO-1 (ab216880), Vimentin (ab11256), Snail (ab180714), MMP-2 (ab37150), GSK-3β (ab73173) and β-actin (ab8227) were purchased from Abcam (Cambridge, UK). Antibody against Ki-67 (AF0198) was purchased from Affinity Biosciences.OH (USA).

***MTT assay***

One thousand cells were seeded into 96-well plates to adhere overnight. Cells were then incubated with indicated treatments for 24 hr, followed by adding 5 mg/ml MTT (Sigma, St. Louis, MO, USA). After incubation at 37 °C for 4 hr, the supernatants were removed, and 100 μl of DMSO were added. The absorbance value at 570 nm was measured using a spectrophotometer (TECAN Infinite M200PRO, Switzerland). Cell viability (%) was normalized to the absorbance value of untreated control cells.

***Hoechst 33342 staining***

Cells were seeded at 1 × 10^4^ cells/ml and cultured for 2 to 4 days until they reached 80% to 90% confluence. The cells were then fixed for 10 min at room temperature with 3% paraformaldehyde, followed by staining with 1 mg/ml Hoechst33342 at room temperature for 5 min. The cells were then washed twice with PBS, examined, and immediately photographed under a fluorescence microscope (OLYMPUS, Japan).

***Colony formation assay***

Two hundred cells were seeded into 100 mm dishes, cultured for 24 hr, and treated with BAI at concentrations of 5 μM, 10 μM, and 25μM for 24 hr. Then, cells were cultured for up to 3 weeks. Colonies (more than 50 cells per colony) were visualized and counted using Giemsa staining.

***Wound healing assay***

The wounds were created by scratching the dish with a micropipette tip. The medium was immediately replaced, and spontaneous cell migration was monitored using an inverted microscope (Nikon, Japan) at 0 hr, 24 hr, and 48 hr. The width of the wound gap was measured in three independent wound sites per group at each time point. 

***Immunofluorescence***

Cells grown in six-well culture slides were fixed with 4% paraformaldehyde for 15 min, permeabilized with 0.5% Triton X-100 (CWBIO, China), and blocked with 3% BSA for 2 hr. Cells were incubated with primary antibody of E-cadherin and Snail at 4 °C overnight, washed three times with PBS, and incubated with Alexa Fluor 488 or Alexa Fluor 568-labeled goat anti-rabbit IgG secondary antibody (Invitrogen) for 2 hr, and then analyzed by a confocal microscope (Leica SP5II Microsystems, Mannheim, Germany).

***Transwell assay***

The Transwell assay used 24-well BD BioCoat Matrigel invasion chambers Millicell (Millipore, MA, US) with 8-μm pore inserts. Cells (5 × 10^4^) were seeded into the upper insert in serum-free media, while media containing 10% FBS was added to the lower chambers as a chemoattractant for 48 hr. The cells were removed from the upper surface of the filter by scraping with a cotton swab. Cells that infiltrated through the filter were fixed and stained with Gemisa. The images were taken with OLYMPUS BX53 (OLYMPUS, Japan). The mean values of the cell number were obtained from three chambers. 

***Quantitative real-time PCR (qRT-PCR)***

MKN-74 and MGC-803 cells were cultured in 6-well plates. After incubation overnight, the cells were treated with 25 μM BAI for 24 hr and 48 hr. Then, the cells were harvested, and total RNA was isolated using a TRIzol extraction kit (Thermo Fisher Scientific, Waltham, MA, USA). cDNA was synthesized from RNA using the PrimeScript^TM^ RT reagent kit (Takara, Dalian, China). qPCR was performed using SYBR Green qPCR Master Mix (Takara, Dalian, China), and the expression of mRNA for *p*12-LOX and GAPDH (internal control) was measured. qPCR was performed using a Mx3000P QPCR System (Agilent Technologies, StrataGene, USA), and the cycling conditions were as follows: 95 °C for 10 min; 40 cycles of 95 °C for 15 sec, 60 °C for 1 min and 72 °C for 40 sec; and a final extension step at 72 °C. The relative mRNA level was calculated using the 2^-ΔΔCt^ method and normalized to that of GAPDH. 

***Western blot***

Cells were harvested and lysed with RIPA buffer containing 1 mM PMSF and a protease inhibitor cocktail (Roche, Germany). The protein concentration was measured using a BCA protein assay kit (Pierce, Rockford, Illinois). Proteins (20 μg/lane) were electrophoresed on 8%-12% SDS polyacrylamide gel and transferred to PVDF membranes (Bio-Rad, USA). Membranes were blocked by incubation in 5% skim milk for 1.5 hr and probed with appropriate antibodies (1:1000) at 4 °C overnight, followed by incubation with secondary antibodies of goat anti-rabbit IgG-HRP and goat anti-mouse IgG-HRP (1:5000) at room temperature for 1.5 hr. After color development, the blots were analyzed quantitatively using a Chemiluminescent and Fluorescent Imaging System (ChemiDoc, BIO-RAD, USA).

***In vivo mouse xenografts***

BALB/c nude mice 4–6 weeks old were purchased from the Changzhou Kavins Experimental Animal Co. Ltd. (Changzhou, China). All mice were housed in specific pathogen-free conditions following the guidelines of the Institutional Animal Care. MGC-803 cells (1×10^6^) were implanted subcutaneously into the underarms of nude mice to establish a tumor model. The mice were randomly divided into two groups (n = 4 per group): the control and BAI groups. The mice received intragastric injections of BAI (50 mg/kg) once daily for three weeks (20,21). The weight of the mice was measured every two days, and caliper measurements were performed every two days to calculate tumor volumes. The animals were sacrificed after three weeks of treatment. A portion of each tumor was excised and placed in formalin, and a portion was fresh-frozen in liquid nitrogen for further analysis.

***Immunohistochemistry staining***

The tissue sections were deparaffinized and rehydrated. The antigen was retrieved by a sodium citrate buffer. This was followed by endogenous peroxidase blocking and incubation in normal goat serum (Zhongshan Jinqiao Biotechnology Co, China) for 20 min; these sections were incubated with p-ERK1/2 and ZO-1 (1:200, Abcam, UK), Ki-67 (1:200, AF0198, Affinity Biosciences.OH. USA), overnight at 4 °C and then with the peroxidase-conjugated secondary antibody (Zhongshan Jinqiao Biotechnology Co, China). Color development was performed with 3,3’-diaminobenzidine (DAB), and slides were counterstained with hematoxylin. Neutral resin sealing was performed. The results of immunohistochemical staining were observed under a microscope (Olympus Tokyo, Japan).

***Statistical analysis***

**Figure 1:**
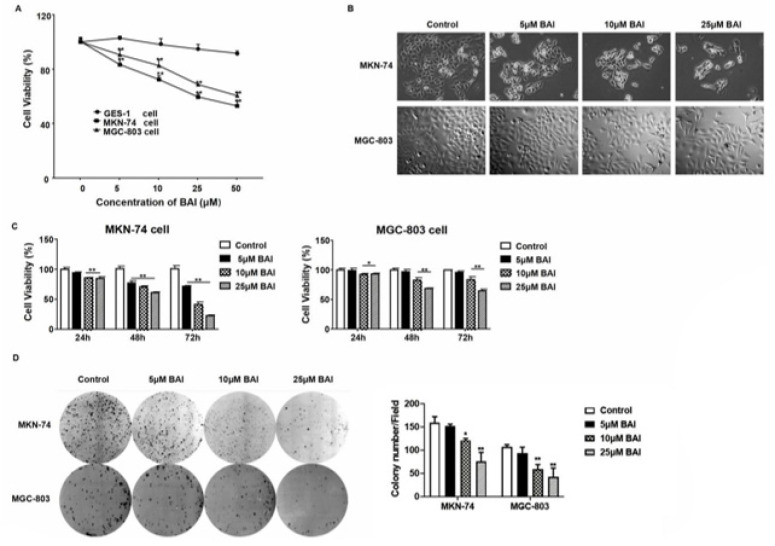
Cytotoxicity of BAI on GES-1, MKN-74, and MGC-803 cells

Statistical analysis was performed using the SPSS 17.0 software (SPSS, Chicago, IL, USA) and GraphPad Prism 6.0 software (GraphPad Software, Inc., San Diego, CA, USA). Experiments were performed in triplicate. Unless otherwise stated, data were presented as mean ± standard deviation (SD). In the MTT experiment, two-way ANOVA was used to compare two factors: concentration and cell type (Figure 1A). A comparison between two independent measurement data sets was conducted using the Student’s *t*-test (Figure 5A, 5B). Comparison of measurement data among multiple groups: One-way ANOVA was used. When all groups were compared with the Control group, the Dunnett-t test was used. The LSD method or Bonferroni method (Repeated Measures) was used when comparing any group. Comparison of body weight in animal experiments: Repeated Measures ANOVA was used (Figure 7A). Survival curves were calculated using the Kaplan-Meier analysis. Probability values were two-tailed, and *P*<0.05 was considered statistically significant.

A. Cell viability of the three cell lines after being treated with different concentrations of BAI, which was measured by MTT assay after 24 hr of treatment. B. The cell morphologies of the MKN-74 and MGC-803 cells, after being treated with different concentrations of BAI, were photographed by interference light microscopy (magnification 200×). C. Viability of MKN-74 and MGC-803 cells after being treated with various concentrations of BAI at different times. D. Results of colony formation assay. Two-way ANOVA, *P<0.05, ** *P*<0.01, compared with untreated cells or control.

BAI: Baicalein; MTT: 3-(4,5-dimethyl-2-thiazolyl)-2,5-diphenyl-2-H-tetrazolium bromide

**Figure 2:**
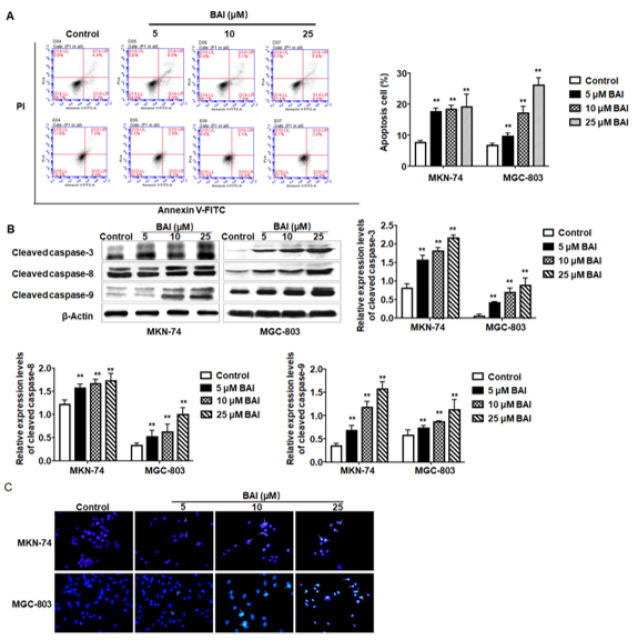
Treatment with BAI induces apoptosis of MKN-74 and MGC-803 cells

A. MKN-74 and MGC-803 cells were exposed to the indicated concentrations of BAI for 48 hr, and the cells were stained with Annexin V-FITC/PI. Apoptotic cells were analyzed by flow cytometry. B. Protein levels of cleaved caspase-3, -8, and -9 were determined by Western blot and quantified by the grey intensity of the protein bands, using β-actin as the internal control. C. Cells were stained with Hoechst33342 and analyzed by fluorescence microscopy. Arrows indicate the apoptotic nuclei. One-way ANOVA, **P< 0.01 compared with the control.

BAI: Baicalein

**Figure 3:**
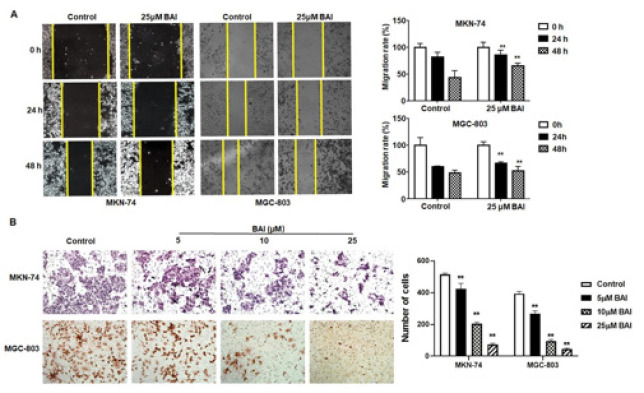
Treatment with BAI inhibits MKN-74 and MGC-803 gastric cancer cell migration and invasion

A. MKN-74 and MGC-803 gastric cancer cells were treated with 25 μM BAI for 24 hr, and then the wounds were made. Photographs were taken at 0 hr, 24 hr, and 48 hr after the wounds were made. The normalized wound area was calculated using a software-based method. B. GES-1, MKN-74, and MGC-803 cells were seeded in Transwell chambers and treated with the indicated concentrations of BAI for 48 hr, and the invasion cells were photographed. One-way ANOVA, **P<0.01, compared with control.

BAI: Baicalein

**Figure 4:**
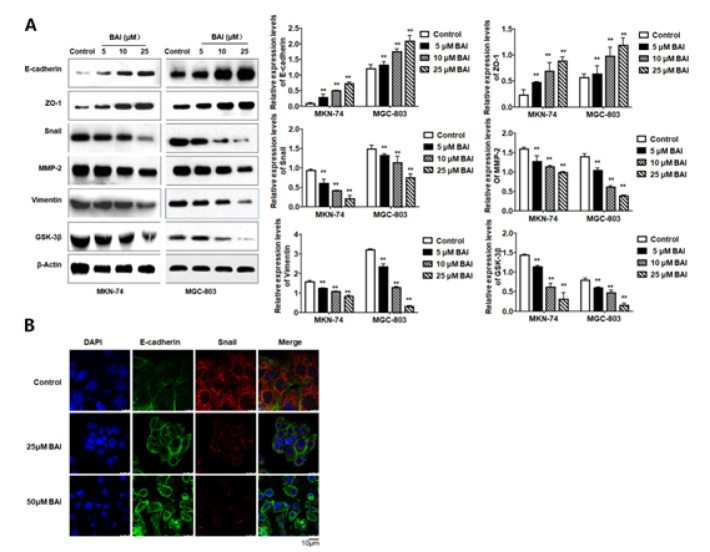
EMT-associated markers in MKN-74 and MGC-803 cells after being treated with BAI

A. The protein expression of EMT-associated markers (E-cadherin, Snail, Vimentin, ZO-1, MMP-2, and GSK-3b) in MKN-74 and MGC-803 cancer cell lines after being treated with various concentrations of BAI for 24 hr, and quantified by the grey intensity of the protein bands, using β-actin as the internal control. One-way ANOVA, **P<0.01, compared with control. B. EMT-associated markers (E-cadherin and Snail) in MKN-74 cells were visualized by immunofluorescence after being treated with varied concentrations of BAI for 24 hr.

BAI: Baicalein

**Figure 5:**
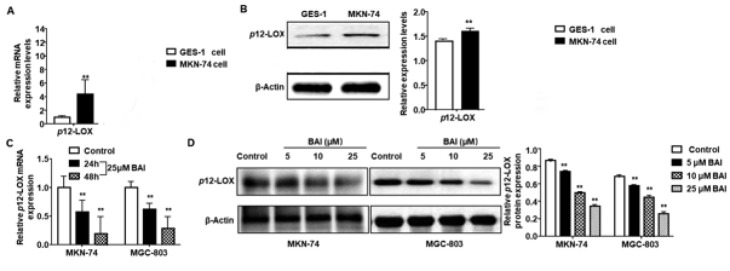
Expressions of *p*12-LOX in cancer and normal gastric cell lines

A. The expression levels of *p*12-LOX mRNA in gastric cancer MKN-74 cell and normal gastric mucosa GES-1 cell lines determined by qPCR, using GAPDH as the internal control. Student’s t-test, **P<0.01, compared with GES-1 cells. B. The expression levels of *p*12-LOX proteins in MKN-74 and GES-1 cell lines were determined by western blot, using b-actin as the internal control. Student’s t-test, **P<0.01, compared with GES-1 cells. C. The mRNA levels of *p*12-LOX in MKN-74 and MGC-803 cells after treatment with 25 μM BAI for 24 hr and 48 hr. D. Protein levels of *p*12-LOX in MKN-74 and MGC-803 cells after being treated with different concentrations of BAI for 48 hr. One-way ANOVA, **P<0.01, compared with control.

BAI: Baicalein

**Figure 6:**
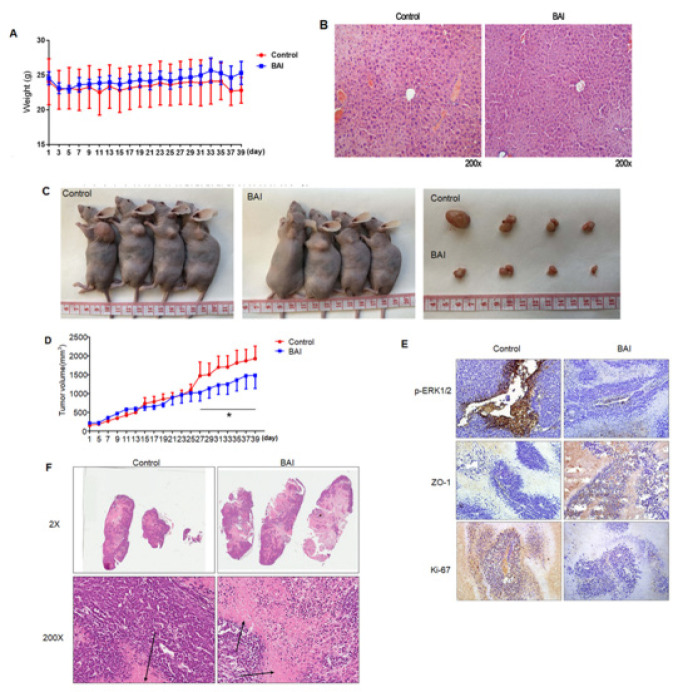
BAI suppresses the expressions of p-MEK1/2 and p-ERK1/2 in gastric cancer cells

The protein expressions of A. t-MEK1/2, p-MEK1/2, t-ERK1/2, and p-ERK1/2 in MKN-74 and MGC-803 cells after being treated with various concentrations of BAI for 24 hr were measured by western blot. The amounts of proteins were quantified by the gray intensity of protein bands, using β-actin as the internal control. One-way ANOVA, **P<0.01, compared with control.

**Figure 7:**
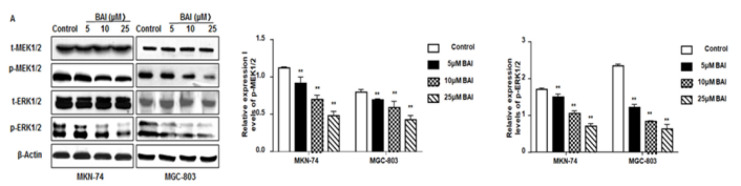
BAI inhibits GC progression via ERK1/2 signaling in a xenograft model

A. Changes in body weight after 40 days of BAI treatment. B. H&E staining of the portal tract to evaluate the toxicity of BAI. No significant changes were found. C. Representative images of nude mice xenografted with MGC-803 cells in the control group and BAI treatment group. D. Changes in tumor volume after 40 days of BAI treatment. Repeated Measures ANOVA, *P<0.05, compared with control. E. IHC staining of the xenograft tumor tissue. IHC staining showed significant inhibition of p-ERK1/2 and Ki-67 expression, but the expression of ZO-1 was significantly increased.

GC: Gastric cancer; BAI: Baicalein

**Figure 8:**
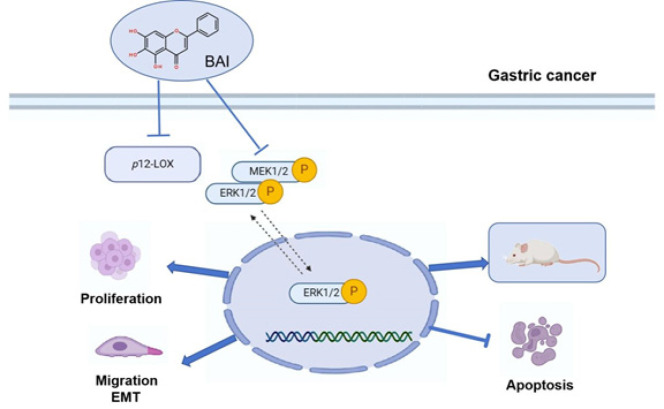
Schematic diagram of baicalein blocked GC proliferation and invasion through modulated *p*12-LOX

GC: Gastric cancer

## Results

***Cytotoxicity of BAI on gastric cancer and normal gastric mucosal epithelial cell lines***

Gastric cancer MKN-74 and MGC-803 cells were divided into the Control and BAI treatment groups. The BAI treatment group was treated with 5, 10, 25, and 50 μM BAI, and the control group received an equal volume of DMSO for 48 hours. The MTT method was used to detect the changes in the proliferation ability of BAI in cells. To clarify the toxic effect of BAI on different cells. The results showed that BAI significantly inhibited the proliferative activity of gastric cancer MKN-74 and MGC-803 cells, but there was no significant change in normal gastric mucosal epithelial GES-1 cells treated with BAI. Further, BAI showed an inhibitory effect on tumor cells but no obvious toxic effect on normal epithelial cells (Figure 1A). The low toxicity of BAI to normal epithelial cells suggests that BAI has certain potential for clinical application. The morphological observation indicated that BAI treatment affected the morphology of MKN-74 and MGC-803 cells. The control cells maintained a fibroblastic, elongated, mesenchymal-like morphology, while cells treated with BAI showed a round, cobblestone, epithelial-like phenotype (Figure 1B). The cell viability and morphological changes showed a dose-dependent manner. In addition, when treated with different doses of BAI, the viability of MKN-74 and MGC-803 cells also decreased in a time-dependent manner (*P*<0.01) (Figure 1C). Similarly, the colony formation capacities were markedly inhibited in the MKN-74 and MGC-803 cells following BAI treatment (Figure 1D). These results indicate that BAI has cytotoxicity against gastric cancer cells. 

***BAI induces apoptosis of MKN-74 and MGC-803 cells***

The effect of BAI on gastric cancer cell apoptosis was further analyzed. Flow cytometric analysis showed that the numbers of apoptotic cells were significantly increased compared with the untreated cells in a dose-dependent manner (*P*<0.01) (Figure 2A). Caspases play pivotal roles in cell apoptosis and are usually considered markers of apoptosis. Western blot showed that the expression levels of cleaved caspase-3, -8, and -9 were all increased significantly (*P*<0.01) (Figure 2B). Additionally, Hoechst 33342 staining showed that BAI-treated MKN-74 and MGC-803 cells exhibited condensed and fragmented nuclei (Figure 2C). These results indicate that treatment with BAI can induce apoptosis in MKN-74 and MGC-803 cells, and the apoptotic cells increased significantly along with the increase in BAI concentration.

***BAI inhibits the migration and invasion ability of MKN-74 and MGC-803 gastric cancer cells***

To determine the effect of BAI on cell migration and invasion, wound healing and Transwell assays were performed. As shown in Figures 3A and 3B, the migration and invasion ability of MKN-74 and MGC-803 cells was significantly inhibited after being treated with BAI, compared with the untreated cells (*P*<0.01). These functional studies highlight the inhibitory effects of BAI on the migration and invasion of gastric cancer cells *in vitro*. 

***BAI inhibits EMT in gastric cancer cells***

EMT-like changes are accompanied by down-regulation of the epithelial marker E-cadherin and up-regulation of mesenchymal markers such as Vimentin and matrix metalloproteinase-2 (MMP-2) (22). Thus, the expressions of EMT-associated proteins were detected by western blot and immunofluorescence. The results revealed that the expression levels of mesenchymal-related proteins, including Vimentin, Snail, GSK-3b, and MMP-2, were lowered in MKN-74 and MGC-803 cells after BAI treatment compared with those in the untreated cells. On the contrary, the protein expression levels of E-cadherin and ZO-1 increased (*P*<0.01) (Figure 4A). The altered expression levels of E-cadherin and Snail were also observed in MKN-74 cells by immunofluorescence. The fluorescence intensity of E-cadherin obviously increased, while that of Snail dramatically decreased (Figure 4B). These results indicate that treatment with BAI can suppress EMT in gastric cancer cells.

***BAI down-regulates the expression of p12-LOX in gastric cancer cells***

To detect the protein and mRNA expression levels of *p*12-LOX in gastric cancer MKN-74 cells and normal gastric mucosa GES-1 cells, qRT-PCR (Figure 5A) and western blot (Figure 5B) were performed. *p*12-LOX were expressed in both cell lines. However, they were significantly higher in gastric cancer MKN-74 cells than those in the normal gastric mucosa GES-1 cells (*P*<0.01). The results suggest that the expressions of *p*12-LOX are up-regulated in the cancerization process of gastric tissue cells. To investigate the effect of BAI on the expression of *p*12-LOX, qPCR and western blot were performed. After treatment with 25 μM BAI for 24 hr and 48 hr, the expressions of *p*12-LOX mRNA in MKN-74 and MGC-803 cells decreased as the treatment time prolonged (*P*<0.01) (Figure 5C). In addition, when MKN-74 and MGC-803 cells were treated with BAI of different concentrations for 24 hr, their protein expression levels of *p*12-LOX decreased in a dose-dependent manner (*P*<0.01) (Figure 5D). The results indicate that BAI can down-regulate the mRNA and protein expressions of *the p*12-LOX gene in a dose **and time-**dependent manner. 

*BAI suppresses p-MEK-1* ***/2*** *and p-ERK1/2 expressions*

To determine the effect of BAI on the MEK/ERK pathway, the protein levels of the key nodes of this pathway, including p-MEK-1/2 and p-ERK1/2, were assessed by western blot. Compared to the control untreated cells, the expressions of p-MEK-1/2 and p-ERK1/2 in MKN-74 cells all decreased as the concentration of BAI increased (*P*<0.01) (Figure 6). The results indicate that BAI may inhibit MEK/ERK pathway activation by reducing the phosphorylation of MEK-1/2 and ERK1/2.

*BAI inhibits GC progression via ERK1/2 signaling in a xenograft model*

To further confirm the anti-tumor effects of BAI *in vivo*, 1×10^6^ MGC-803 cells were subcutaneously transplanted into nude mice (n=4 biologically independent mice per group). Seven days after cancer cell injection, BAI at a 50 mg/kg dose was administered by intragastric administration every 24 hr for 30 days. The mice were sacrificed after 40 days and the results showed there were no significant changes in body weight (Figure 7A). Hematoxylin and eosin (H&E) staining of the portal tract was undertaken to evaluate the toxicity of BAI and revealed no significant changes (Figure 7B), indicating that BAI did little damage at a dose of 50 mg/kg. Compared with the control group, the tumor size of mice in the BAI group was significantly inhibited (Figure 7C). Tumor volumes were significantly reduced compared with the control group (Figure 7D). Next, we applied IHC staining to the xenograft tumor tissue. IHC staining showed a significant inhibition of p-ERK1/2 and Ki-67 expression compared with the control group, but the expression of ZO-1 significantly increased in Figure 7E. Thus, our data indicated that BAI inhibited GC progression via ERK1/2 signaling *in vivo*.

## Discussion

**The occurrence and development of gastric cancer are very complex, and the underlying mechanism has not been fully elucidated yet. Therefore, studying the molecular mechanism and developing targeted therapy for gastric cancer is very significant. BAI, a selective inhibitor of** ***p*** **12-LOX, has effects of anti-inflammation, anti-virus, antitumor immunity and anti-oxidation** (23,24)**. A previous study has illustrated that BAI can inhibit the proliferation and inhibit invasion and metastasis of non-small-cell lung cancer** (25)**. This study found that BAI effectively inhibited the proliferation and induced apoptosis of both MKN-74 and MGC-803 cells. It also inhibited the occurrence of EMT, down-regulated *p*12-LOX, proteins in the MAPK/ERK pathways. Moreover, *in vivo *xenograft study demonstrated that BAI inhibited tumor growth. Our findings confirm the inhibitory effect of BAI on gastric cancer cell growth both *in vitro* and *in vivo*, and indicate that* p*12-LOX may be used as the new molecular target for gastric cancer treatment.**

**LOX plays an important role in tumor formation and the proliferation and migration of tumor cells** (26)**. It has been reported that *p*12-LOX is over-expressed in different cancers, including gastric, colorectal, and kidney cancer, and participates in the occurrence and development of tumors** (27)**. Our previous study reported that BAI inhibited the expression of *p*12-LOX mRNA and protein in mice epidermal JB6 P+ cells and inhibited JB6 P+ cell proliferation in a dose- and time-dependent manner** (11)**. In this study, the treatment with BAI caused significant MKN-74 and MGC-803 cell growth arrest both *in vitro* and *in vivo*. The mRNA and protein expression levels of *p*12-LOX were significantly higher in the gastric cancer MKN-74 and MGC-803 cells than those in the normal human gastric mucosal epithelial GES-1 cells, suggesting that the high expression of *p*12-LOX genes may be associated with the occurrence of gastric cancer. The treatment with BAI showed a significant inhibitory effect on the growth of MKN-74 and MGC-803 cells in a dose- and time-dependent manner. When treated with BAI, the mRNA and protein levels of *p*12-LOX in MKN-74 and MGC-803 cells decreased, suggesting that BAI can down-regulate the expression of the *p*12-LOX gene. These results indicate that the inhibited proliferation of MKN-74 and MGC-803 cells by BAI may be associated with the reduced expressions of *p*12-LOX genes in a dose- and time-dependent manner. **

**As well known, detecting active caspases in cells and tissues is an important method for measuring apoptosis induced by a wide variety of apoptotic signals** (28)**. Some Chinese herbs have been reported to promote the apoptosis of tumor cells, Zhang *et al*. reported that convallatoxin potentiates apoptosis and the expression of cleaved caspase-3 protein in HCT116 cells** (29)**. Additionally, after exposure to different concentrations of BAI, the number of apoptotic MKN-74 and MGC-803 cells significantly increased in a dose-dependent manner. In the meantime, the expressions of cleaved caspase-3, -8, and -9 were up-regulated after BAI treatment.** These results indicate that BAI inhibits gastric cancer cell proliferation by promoting cell apoptosis. 

**EMT is the process of epithelial cells transforming into mesenchymal phenotype during various physiological as well as pathological processes, such as embryonic development, wound healing, and tumor progression, by which epithelial cells reduce their adhesions and become migratory and invasive** (30, 31)**. Hence, EMT-induced tumor cell dissemination and drug ineffectiveness in cancer therapy have been intensively studied. Recent studies have suggested that inhibition of the JNK/ERK and PI3K/AKT pathways can suppress EMT progression** (32)**. Ma *et al*. reported that BAI suppressed EMT in breast cancer** (12)**.** Another study** also reported that BAI inhibited EMT in MDA-MB-231 cells** (33)**. ERK1/2 signaling pathways are classical signaling pathways in tumor research, which are involved in regulating cell proliferation, migration, apoptosis, autophagy, and other biological functions** (34)**. Taken together, BAI can effectively suppress gastric cancer malignant transformation by driving cancer cells toward an epithelial state. Luo *et al*. **(35)** reported that MEK/Erk1/2 pathway inactivation and BAI treatments could induce apoptosis, which is associated with the mitochondria apoptosis pathway. Moreover, reports have also shown that MEK/Erk1/2 pathway inactivation and *p*12-LOX inhibitor treatments suppress tumor angiogenesis** (36)**. Therefore, further studies are needed to explore the effects of BAI on gastric cancer angiogenesis and autophagy. We next generated a xenograft model to confirm our findings further. BAI treatment significantly down-regulated the relative volume of subcutaneously transplanted tumors, and IHC staining of tumor tissue revealed that p-ERK1/2 was inhibited. **

**In recent years, the combination of drugs has been a common practice for enhancing the efficiency of drug treatment, but the selection of the optimal combination and optimal dose remains a matter of trial and error (** 37 **). Plant-derived bioactive compounds are a prominent alternative medicinal approach for the reduction of chemotherapy-associated side effects (** 38 **). A study found that baicalein enhanced the cisplatin sensitivity of SGC-7901/cisplatin gastric cancer cells by inducing apoptosis and autophagy via Akt/mTOR and Nrf2/Keap 1 pathways (** 39 **). Another study detected that inhibition of glycolysis via regulation of the PTEN/Akt/HIF-1α signaling pathway may be one of the mechanisms whereby baicalein reverses 5-FU resistance in cancer cells under hypoxia (40). These papers revealed that new strategic approaches and **future perspectives **for cancer treatment, including the combination therapy of cisplatin, 5-fluorouracil, and baicalein, have been evaluated with some degree of success.**

## Conclusion

**To summarize, BAI significantly inhibited the proliferation and induced apoptosis of gastric cancer cells**,** probably by inactivating MEK/ERK1/2 pathways. Additionally, BAI treatment could suppress gastric cancer cell migration **and EMT in vitro and in vivo (Figure 8). **These results highlight the therapeutic strategy using BAI for the treatment of patients with advanced gastric cancer.**
